# Copper-induced activation of TRPs and VDCCs triggers a calcium signature response regulating gene expression in *Ectocarpus siliculosus*

**DOI:** 10.7717/peerj.4556

**Published:** 2018-04-16

**Authors:** Alberto González, Claudio A. Sáez, Alejandra Moenne

**Affiliations:** 1 Laboratory of Marine Biotechnology, Faculty of Chemistry and Biology, Universidad de Santiago de Chile, Santiago, Región Metropolitana, Chile; 2 Laboratory of Costal Environmental Research, Center of Advanced Studies, Universidad de Playa Ancha, Viña del Mar, Valparaíso, Chile

**Keywords:** Calcium, Copper, Gene expression, Voltage-dependent calcium channels, Transient receptor potential channels

## Abstract

In certain multicellular photoautotrophs, such as plants and green macroalgae, it has been demonstrated that calcium signaling importantly mediates tolerance to copper excess. However, there is no information in brown macroalgae, which are phylogenetically distant from green algae and plants. We have previously shown that chronic copper levels (2.5 μM) activate transient receptor potential (TRP) channels in the model brown macroalga *Ectocarpus siliculosus*, allowing extracellular calcium entry at 13, 29, 39 and 51 min. Here, we showed that intracellular calcium increases also occurred at 3 and 5 h of exposure; these increases were inhibited by antagonists of voltage-dependent calcium channels (VDCCs); a chelating agent of extracellular calcium; an antagonist of endoplasmic reticulum (ER) ATPase; and antagonists of cADPR-, NAADP- and IP_3_-dependent calcium channels. Thus, copper activates VDCCs allowing extracellular calcium entry and intracellular calcium release from the ER via cADPR-, IP_3_- and NAADP-dependent channels. Furthermore, the level of transcripts encoding a phytochelatin synthase (PS) and a metallothionein (MT) were analyzed in the alga exposed to 2.5 μM copper from 3 to 24 h. The level of *ps* and *mt* transcripts increased until 24 h and these increases were inhibited by antagonists of calmodulins (CaMs), calcineurin B-like proteins (CBLs) and calcium-dependent protein kinases (CDPKs). Finally, activation of VDCC was inhibited by a mixture of TRP antagonists and by inhibitors of protein kinases. Thus, copper-mediated activation of TRPs triggers VDCCs via protein kinases, allowing extracellular calcium entry and intracellular calcium release from ER that, in turn, activate CaMs, CBLs and CDPKs increasing expression of PS and MT encoding genes in *E. siliculosus*.

## Introduction

Calcium signaling is a well-known mechanism of regulation of physiological performance in plants, a complex phenomenon in which the changes in magnitude, localization and time-scale of calcium signals, defined as “calcium signature,” determine responses of plant cells to environmental stimuli (Kudla, Batistic & Hashimoto, 2010; Stael et al., 2012; Kudla et al., 2018). It has been observed that calcium signals start in microdomains of cellular membranes, specifically in sensors and receptors associated with calcium channels, such as transient receptor potential (TRP) and voltage-dependent calcium channels (VDCCs) (Rizzuto & Pozzan, 2006). TRP channels are ionotropic non-specific cation channels permeable to calcium; these have been observed mammals, insects, nematodes and macroalgae, but not in plants (Nilius & Flockerzi, 2014; Madrid & Bacigalupo, 2015; Gómez et al., 2015). On the other hand, VDCCs are ionotropic calcium-selective channels present in vertebrates, invertebrates, plants and algae (White, 2000; Catterall, 2011). Despite the contribution of extracellular calcium, cellular organelles such as the vacuole, mitochondria and chloroplasts, also participate in calcium release at the intracellular level (González et al., 2012a; Kudla et al., 2018). In regard with the calcium transduction pathways, a vast amount of calcium-responsive proteins have been discovered, counting the presence of several sets of calmodulins (CaMs) and calmodulin-like proteins (Zhu et al., 2015), calcium-dependent protein kinases (CDPKs) (Wernimont et al., 2010), calcineurin B-like proteins (CBLs) (Batistic & Kudla, 2009) and CBL-interacting protein kinases (CIPKs) (D’Angelo et al., 2006). These proteins are able to activate transcription factors regulating gene expression, in order to fulfil cellular processes as nutrient sensing and acquisition (Straub, Ludewig & Neuhäuser, 2017), and tolerance to abiotic stressors (Kilian et al., 2007).

Although most of the information regarding calcium signaling in multicellular photoautotrophs is related to vascular plants, there are some records in other photosynthetic relevant habitat-forming organisms, such as marine macroalgae. For instance, it has been demonstrated the involvement of calcium and CaMs in the development of zygotes of the brown macroalga *Pelvetia compressa* (Pu & Robinson, 1998). More recently, it has been determined that calcium waves mediate polarized growth in rhizoids of the brown seaweed *Fucus vesiculosus* (Coelho et al., 2002). In spite of this information, there is a lack of studies demonstrating the potential involvement of calcium signaling in processes determining tolerance to abiotic stressors in macroalgae, for example, metal pollution. Almost the only records available on calcium signaling regarding a tolerance response are associated with the green macroalga *Ulva compressa* (see Moenne, González & Sáez, 2016). In this context, it has been shown that copper excess on *U. compressa* activates TRP channels leading to extracellular calcium entry and intracellular calcium increases at 4, 8 and 12 min of exposure (Gómez et al., 2015). Moreover, increments in intracellular calcium were observed at 2, 3 and 12 h of copper exposure, which involved the activation of VDCCs allowing extracellular calcium entry and intracellular calcium release from the endoplasmic reticulum (ER) (González et al., 2012b). Calcium release from the ER also involved the activation of cADPR-, NAADP- and IP_3_-dependent calcium channels (González et al., 2010a, 2012b). Moreover, the increase in intracellular calcium activates CaMs and CDPKs that, in turn, lead to upregulation of antioxidant enzymes superoxide dismutase (SOD), ascorbate peroxidase (AP), glutathione reductase (GR) and peroxiredoxin (PRX), and also metallothioneins (MTs), demonstrating the role of calcium signaling in metal tolerance processes related to the antioxidant metabolism and metal tolerance (González et al., 2012a; Laporte et al., 2016). Finally, it was observed that copper allows extracellular copper ions entry leading to membrane depolarization events that occur at 1, 2, 4, 8, 12, 80 and 86 min, as well as at 5 and 9 h of exposure (Gómez et al., 2015, 2016). Despite the available information in green macroalgae, these cannot be directly extrapolated to other seaweeds; indeed, it is known the long phylogenetic distance between red (Rhodophyta) and green (Chlorophyta) with brown (Heterokonta) macroalgae (Cock et al., 2010).

Although copper is an essential metal, beyond certain threshold concentrations it can become toxic for marine organisms, also for brown macroalgae. Different strains of the brown macroalga *Ectocarpus siliculosus* have demonstrated to tolerate chronic copper exposure of up to 2.4 μM, manifested in terms of growth, cellular integrity and photosynthetic performance (Ritter et al., 2010; Roncarati et al., 2015). *E. siliculosus* mechanisms to withstand copper excess have been observed to be importantly mediated by cell wall chelation as an exclusion strategy, and the production of intracellular metal-chelating peptides, as glutathione (GSH) and phytochelatins (PCs) (Roncarati et al., 2015). In addition, it has been observed that copper-induced oxidative stress and damage in *E. siliculosus* is counteracted through the glutathione–ascorbate (Foyer–Halliwell–Asada) cycle, which involves maintaining the equilibrium among reduced and oxidized forms of glutathione (GSH/GSSG) and ascorbate (ASC/DHA/MDHA), and enhanced activities and expression of the enzymes as GR, AP, SOD and catalase (CAT) (Sáez et al., 2015a, 2015b, 2015c). It is important to mention that the genome of *E. siliculosus* has been already published (Cock et al., 2010), providing unprecedented possibilities to deepen on aspects currently unexplored regarding metal-stress metabolism in brown macroalgae; for instance, elucidating the potential involvement of calcium signaling.

In this work, calcium levels were analyzed in *E. siliculosus* up to 12 h of chronic copper exposure. In this context, the nature of channels allowing calcium entry was also studied. Furthermore, the potential entry of extracellular calcium and intracellular calcium release were investigated. Finally, the involvement of the signaling pathways mediated by CaMs, CBLs and CDPKs were studied, also to address their eventual contribution for the regulation of gene expression; the latter was achieved through the measurement of gene-transcripts encoding enzymes participating in the syntheses of the metal-chelating PCs and MTs.

## Materials and Methods

### Algal culture and experimental design

*Ectocarpus siliculosus* strain Es524 (CCAP 1310/333) was cultivated in vitro using 10 L polycarbonate bottles containing sterile seawater enriched with Provasoli nutrients (Provasoli & Carlucci, 1974), at 14 °C, 70 μmol^−1^ s^−1^ PAR, and 16:8 h light/dark cycles. Constant filtered air bubbling was provided to avoid O_2_ depletion. The strain Es524 was chosen as is a representative of an *E. siliculosus* population commonly exposed to copper pollution (see Ritter et al., 2010; Sáez et al., 2015a, 2015b, 2015c); thus, its responses should be able to provide insights on the development of tolerance strategies in brown macroalgae to withstand copper excess in the natural environment.

Inhibitors were purchased from Tocris-Bioscience (Bristol, UK). Specific inhibitors of VDCCs were verapamil, nifedipine and diltiazem (Triggle, 2006) calcium channels were: ryanodine, and inhibitor of cADPR-dependent channels (Meissner, 1986); ned-19, an inhibitor of NAADP-dependent channels (Naylor et al., 2009); and xestospongin C, an inhibitor of IP_3_-dependent channels (Vassilev et al., 2001). Inhibitors of calcium-dependent signaling proteins were: W-7, an inhibitor of CaMs (Hidaka et al., 1981); FK506, an inhibitor of CBL (Liu et al., 1991), and staurosporine, an inhibitor of CDPKs (Tanramluk et al., 2009). Inhibitors of TRP channels were: HC030031, an inhibitor of TRPA1 (Eid et al., 2008); ML204, an inhibitor of TRPC4 and TRPC5 (Miller et al., 2011); M8B, an inhibitor of TRPM8 (Almeida et al., 2012); capsazepin (CPZ), an inhibitor of TRPV1 (Maggi et al., 1993). Inhibitors of protein kinase were: KN62, an inhibitor of calcium/calmodulin-dependent kinases (CaMK) (Tokumitsu et al., 1990); chelerythrine, an inhibitor of calcium and diacylglycerol-dependent protein kinase C (PKC) (Herbert et al., 1990); K5720, an inhibitor of cAMP-dependent protein kinase A (PKA) (Kase et al., 1987); and KT5823, an inhibitor of cGMP-dependent protein kinase G (PKG) (Butt et al., 1995).

The alga was cultivated with 2.5 μM of nominal copper (CuCl_2_; Merck, Darmstadt, Germany) and the level of intracellular calcium was detected for 12 h using confocal microscopy. To detect the nature of channels involved in calcium increases, the alga was pre-incubated with 250 nM of VDCC inhibitors for 1 h before copper exposure. After copper was added, the levels of intracellular calcium were detected. To determine whether VDCC allows extracellular calcium entry, the alga was incubated in 0.5 mL of autoclaved seawater containing 10 mM of the non-specific calcium chelator egtazic acid (EGTA) and 2.5 μM copper; the levels of intracellular calcium were observed. It is important to mention that EGTA has been used as a calcium chelator agent for similar investigations aiming to disclose calcium signaling mechanisms in organisms such as animals, plants and algae (Johnson & Dufault, 1993; Perfus-Barbeoch et al., 2002; Hung et al., 2005; Gómez et al., 2015). To determine whether intracellular calcium is released from the ER, the alga was incubated with 250 nM thapsigargin for 30 min and 2.5 μM copper; intracellular calcium was followed up. To determine the nature of calcium channels allowing calcium release from the ER, the alga was cultivated with 250 nM of inhibitors of cADPR-, NAADP- and IP_3_-dependent channel, and with 2.5 μM copper; intracellular calcium was then analyzed.

To assess gene expression associated with metal-chelating metabolites, the alga was cultivated for up to 24 h with 2.5 μM copper. The levels of phytochelatin synthase and metallothionein transcripts (*ps* and *mt*, respectively) were detected using qRT-PCR (see details below). To analyze the involvement of proteins associated with calcium signaling in the activation of gene expression, the alga under 2.5 μM copper was incubated also with 100 nM of inhibitors of CaMs, CBLs and CDPKs (details on inhibitors above); the level of *ps* and *mt* transcripts was determined for up to 24 h. To detect whether TRPs and protein kinases participate in the activation of VDCCs, 100 nM of each TRP inhibitor (see above) or 100 nM of inhibitors of protein kinases (see above) were added for 30 min; then, 2.5 μM copper were added and intracellular calcium increases known to occur at 3 and 9 h were observed. These time-peaks were observed to address for environmentally representative responses in short-term pollution events. To determine the involvement of TRP and VDCC activation in the increase of gene expression, the alga was incubated with 2.5 μM copper and 100 nM of each TRPs inhibitor, or with 2.5 μM copper and 250 nM of VDCCs inhibitors; the level of *ps* and *mt* transcripts were determined at 12 h of copper exposure. It is important to mention that TRP inhibitors were added 10 min before and 60 min after copper addition, respectively, considering that TRP activations occur at 13, 29, 39 and 51 min of copper exposure in *E. siliculosus* (González et al., 2018).

### Detection of intracellular calcium by confocal microscopy

Detection of calcium was performed as described in Gómez et al. (2015, 2016). Algae were gently removed from the culture media and incubated in seawater containing 20 μM Fluo-3AM with 1% DMSO (Molecular Probes, Invitrogen, Eugene, OR, USA) in agitation during 30 min at room temperature. Algae were washed three times in filtered seawater to remove fluorophore excess. The green fluorescence of Fluo 3 was visualized in each filament by confocal microscopy using an Axiovert 100 confocal microscope (Carl Zeiss, Oberkochen, Germany), an emission wavelength of 488 nm produced by an argon laser and a filter of 505–530 nm. The intensity of Fluo-3 green fluorescence was quantified in five cells from each sample in triplicates, on a surface area of 100 μm width by 100 μm length, using LSM510 software of the confocal microscope. Red fluorescence of chloroplast was used for helping focusing the sample and verifying cell integrity (Fig. S1). The fluorescence intensity was expressed as the ratio of change in fluorescence and initial fluorescence.

### Purification of total RNA

RNA extraction was performed with modifications to Greco et al. (2014). Briefly, 500 mg of fresh tissue were pulverized with liquid nitrogen, homogenized in 2 mL of extraction buffer (100 mM Tris–HCl pH 9.5, 150 mM NaCl, 5 mM DTT, 1% sarcosyl), and centrifuged for 20 min at 14,000 rpm. The supernatant was mixed with 0.1 vol of pure ethanol, 0.25 vol potassium acetate (3 M, pH 4.8) and 1 vol chlorophorm/isoamyl alcohol (24:1), mixed for 30 min at 4 °C and centrifuged for 20 min at 14,000 rpm. The aqueous phase was mixed with 0.3 vol ethanol and 1 vol chloroform, mixed for 20 min at 4 °C, and centrifuged for 20 min at 14,000 rpm. The aqueous phase was mixed with 0.1 vol sodium acetate (3 M, pH 5.2), 0.8 vol isopropanol and 1% 2-mercaptoethanol, and stored overnight at −20 °C to precipitate RNA. The mixture was centrifuged at 14,000 rpm for 20 min, the supernatant was removed and the pellet dried using a speed-vac centrifuge BSK-2 (Biobase, Jinan, China). The RNA was further purified using DNAse 1 kit (Sigma-Aldrich, St. Louis, MO, USA) and its integrity was determined using the absorbance ratio A260/A280 according to the manufacturer’s instructions.

### Quantification of antioxidant enzymes transcript levels and treatments

Reverse transcription was performed with 100 ng RNA, using AffinityScript qPCR cDNA synthesis kit containing poly-A primers (Agilent, La Jolla, CA, USA). qPCR was performed using Brilliant III Ultra-Fast SYBR Green QPCR Master Mix (Agilent, La Jolla, CA, USA) using a real time thermocycler Agilent AriaMX platform, programed with 40 cycles of 95 °C for 5 s, 58 °C for 10 s and 60 °C for 10 s. Primers for qPCR were: Phytochelatin synthase (Gene ID Ec-14_005100.2), *ps* forward 5′-CCG ATA CTG TGG GAA GCG AT-3′, *ps* reverse 5′-TTC ACC CAC GAT GCA ACC TT-3′, product size 155 bp, melting temperature 91 °C; Metallothionein (Gene ID Ec-20_001230.1), *mt* forward 5′-CTG TGG GTC GTC GTG CTC T-3′, *mt* reverse 5′-GAT CCG CAG TTG CAG TTG TCC-3′, product size 120 bp, melting temperature 93 °C; β-tubulin was used as housekeeping gene since the level of transcript did not change in response to copper excess, and also considering it has been proposed as such before (Le Bail et al., 2008). Primers used to amplify β-tubulin (Ec-01_004660.1) transcripts were: *tub* forward 5′-TGA TGT TCC GAG GGC GAA TG-3′, and *tub* reverse 5′-GTG TTA CCC ACG AAG GTG GT-3′, product size 169 bp, melting temperature 91 °C. The relative level of transcripts was calculated using the 2^−ΔΔCT^ method (Livak & Schmittgen, 2001).

### Statistical analyses

Significant differences were calculated with one-way ANOVA at 95% confidence interval, using a posthoc Tukey’s test, previous confirmation of requirements of normality and homogeneity of variance using Statgraphics Centurion XVI (StatPoint Technologies Inc., Warrenton, VA, USA) statistical program. Analyses were conducted on three independent replicates. Statistical analysis for qPCR are presented in Tables S1–S4.

## Results

### Copper-mediated activation of VDCCs and calcium release from ER

**Figure 1:**
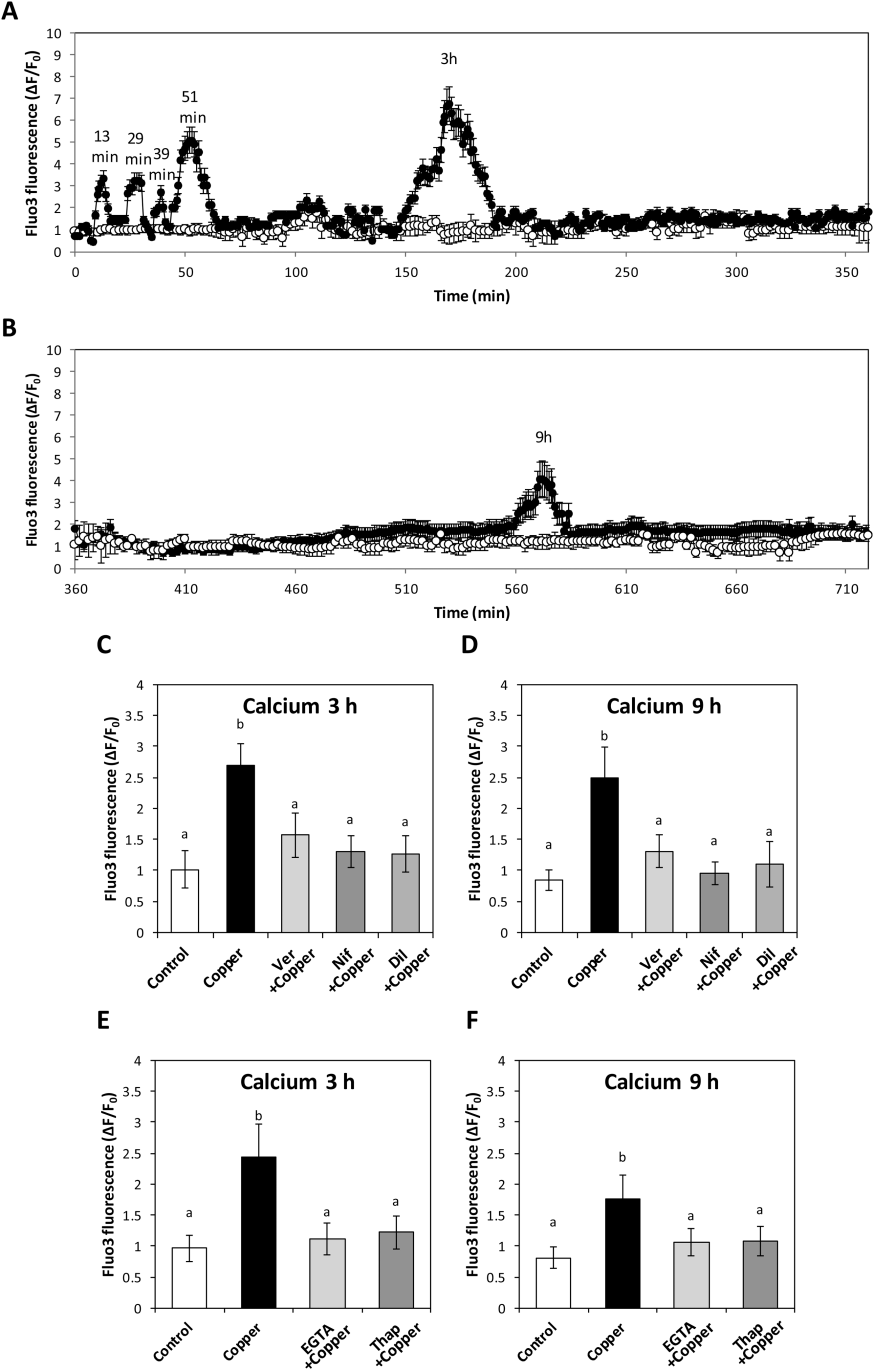
Level of intracellular calcium in *E. siliculosus* cultivated with or without copper excess from 0 to 12 h, and voltage-dependent calcium channels involved with extracellular calcium entry. Level of intracellular calcium in *E. siliculosus* cultivated in seawater without copper addition (empty circles) and with 2.5 μM copper (black circles) for up to 350 min (A), and from 360 to 720 min (B). To assess VDCCs at 3 h (C) and 9 h (D) of copper exposure, treatments were: without copper (control); with 2.5 μM copper (copper); with copper and 250 nM of verapamil (Ver); with copper and 250 nM nifedpine (Nif); and with copper and 250 nM diltiazem (Dil). To address the nature of VDCCs, levels of intracellular calcium in the alga under copper excess at 3 h (E) and 9 h (F) were measured with the treatments: without copper (control); with 2.5 μM copper (copper); with copper and 1 mM EGTA; and with copper and 250 nM thapsigargin (Thap). The level of intracellular calcium is expressed as the difference among initial and final fluorescence intensity of Fluo 3 normalized to initial intensity of Fluo3. Symbols and bars represent mean values of three independent experiments ± SD.

In order to detect intracellular calcium levels in *E. siliculosus* exposed copper excess, the alga was incubated with 2.5 μM copper for 12 h. Calcium increases were detected with peaks at 13, 29, 39 and 51 min of copper exposure (Fig. 1A). In addition, increases in intracellular calcium were detected with maximum peaks at 3 and 9 h of copper exposure (Figs. 1A and 1B). In order to determine the nature of channels involved in calcium increases at 3 and 9 h, the alga was incubated with 2.5 μM copper and 250 nM of inhibitors of VDCCs, verapamil, nifedipine and diltiazem; intracellular calcium was detected. Increases in intracellular calcium detected at 3 and 9 h of copper exposure were completely inhibited by VDCC antagonists (Figs. 1C and 1D). In order to determine whether copper-induced activation of VDCC allows extracellular calcium entry, the alga was incubated with 10 mM EGTA. Moreover, to address whether increase in intracellular calcium mediated by copper is due to the release from ER, the alga was also cultivated with 100 nM thapsigargin, an inhibitor the ER calcium ATPase; intracellular calcium levels were recorded. EGTA and thapsigargin completely inhibited the increase in intracellular calcium observed at 3 and 9 h of copper exposure (Figs. 1E and 1F).

### Copper-induced calcium release from ER involves activation of cADPR-, NAADP- and IP_3_-dependent calcium channels

**Figure 2:**
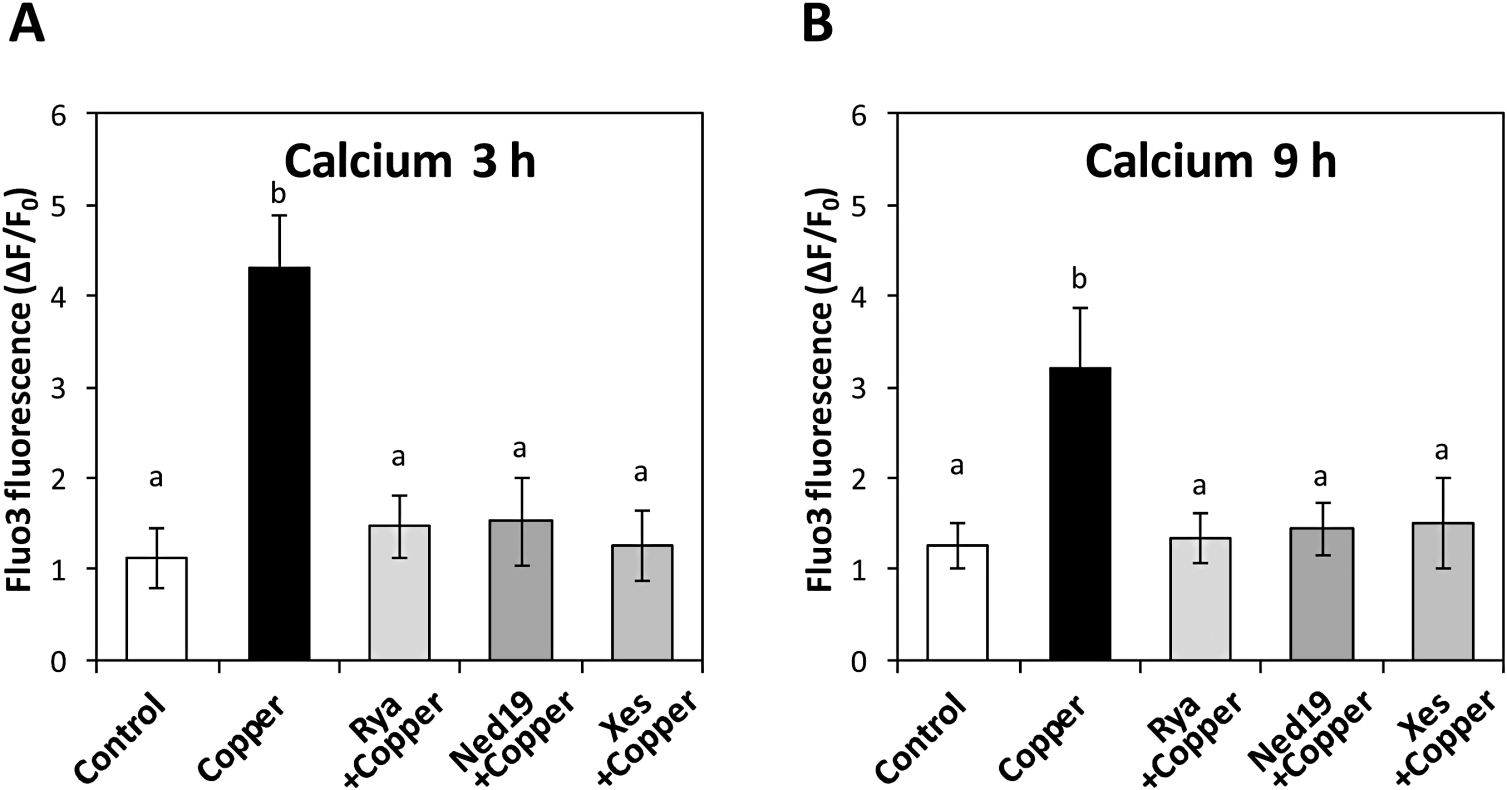
Endoplasmic reticulum calcium channels involved with intracellular calcium release in response to copper excess. To study endoplasmic reticulum calcium channels, level of intracellular calcium in *E. siliculosus* under copper excess at 3 h (A) and 9 h (B) were studied with the treatments: without copper addition (control); with 2.5 μM copper (copper); with copper and 100 nM of ryanodine (Rya); with copper and 100 nM ned-19; and with copper and 100 nM xestospongin C (Xes). The level of intracellular calcium is expressed as the difference among initial and final fluorescence intensity of Fluo 3 normalized to initial intensity of Fluo3. Bars represent mean values of three independent experiments ± SD.

To identify the nature of channels involved in calcium release from the ER, the alga was incubated under copper excess with: ryanodine, an inhibitor of cADPR-dependent calcium channels; ned-19, an inhibitor of NAADP-dependent calcium channels; and xestospongin C, an inhibitor of IP_3_-dependent calcium channels. The levels of intracellular calcium were detected at 3 and 9 h of copper exposure. The inhibitors of ER calcium channels completely inhibited intracellular calcium increases observed at 3 and 9 h of copper exposure (Figs. 2A and 2B).

### Copper-induced increases in intracellular calcium and involvement of CaMs, CBLs and CDPKs on the expression of proteins with roles in metal chelation

**Figure 3:**
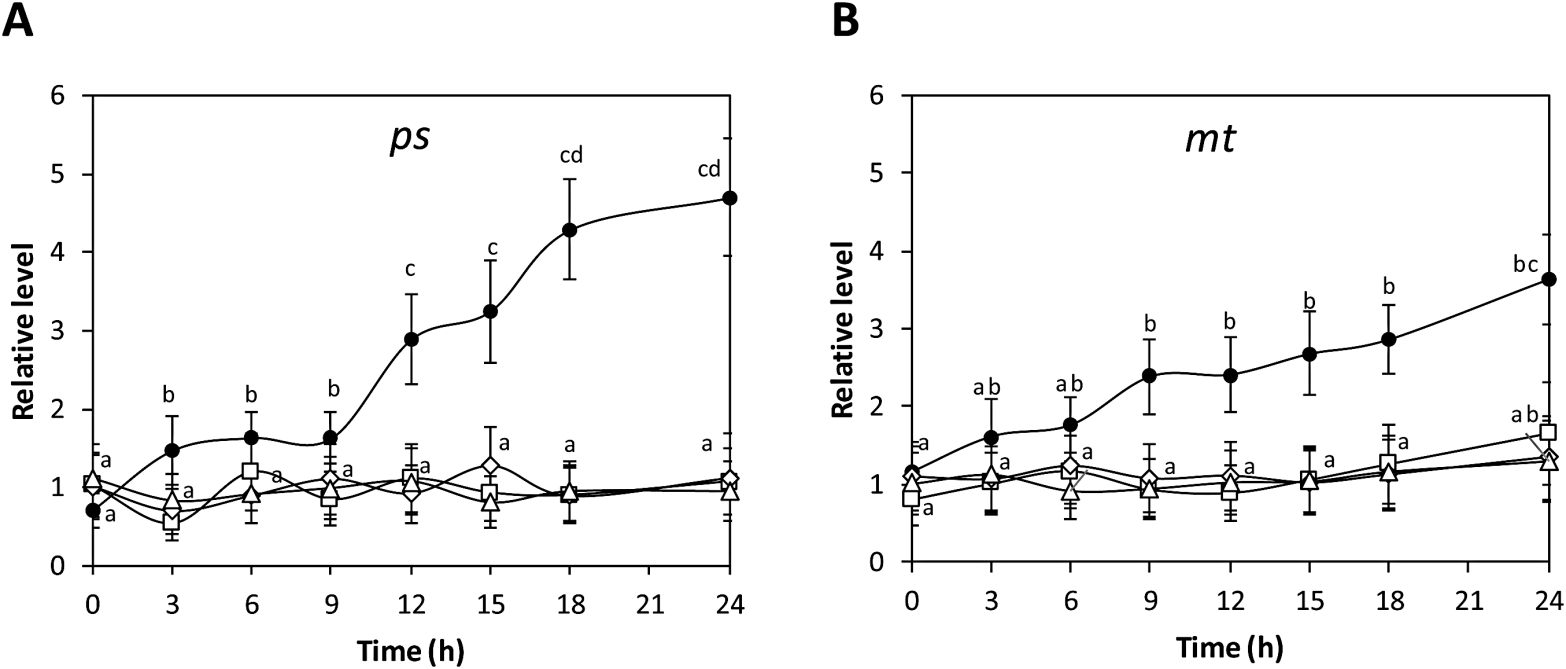
Regulation of copper tolerance related gene expression by calcium-dependent signaling proteins. To study the role of calcium-dependent signaling proteins in regulating the expression of enzymes involved in metal chelator syntheses, level of transcripts *ps* encoding phytochelatin synthase (A) and *mt* encoding metallothionein (B) in *E. siliculosus* under copper excess were detected for up to 24 h with the treatments: 2.5 μM copper (black circles) and with 250 nM W-7 (empty diamonds); with copper and 250 nM FK506 (empty triangles); and with copper and 250 nM staurosporine (empty squares). The relative level of transcripts is expressed as 2^−ΔΔCT^ and time in hours (h). Symbols represent the mean value of three independent experiments ± SD.

To study whether copper-induced greater intracellular calcium mediated also an increase in transcript levels involved in the syntheses of metal chelators, the alga was incubated with 250 nM of inhibitors of calcium signaling proteins such as: W-7, an inhibitor of CaMs; FK506, an inhibitor of calcineurin-like proteins (CBLs), and staurosporine, an inhibitor of CDPKs. Then, the levels of transcripts encoding phytochelatin synthase (*ps*) and metallothionein (*mt*) were quantified for up to 24 h. The level of transcripts of *ps* and *mt* increased maintaining almost a linear pattern upon time of exposure h (Figs. 3A and 3B). In addition, inhibitors of CaMs, CBLs and CDPKs completely inhibited the increase in *ps* and *mt* transcript levels (Figs. 3A and 3B).

### Copper-induced activation of TRPs is involved in VDCCs activation

**Figure 4:**
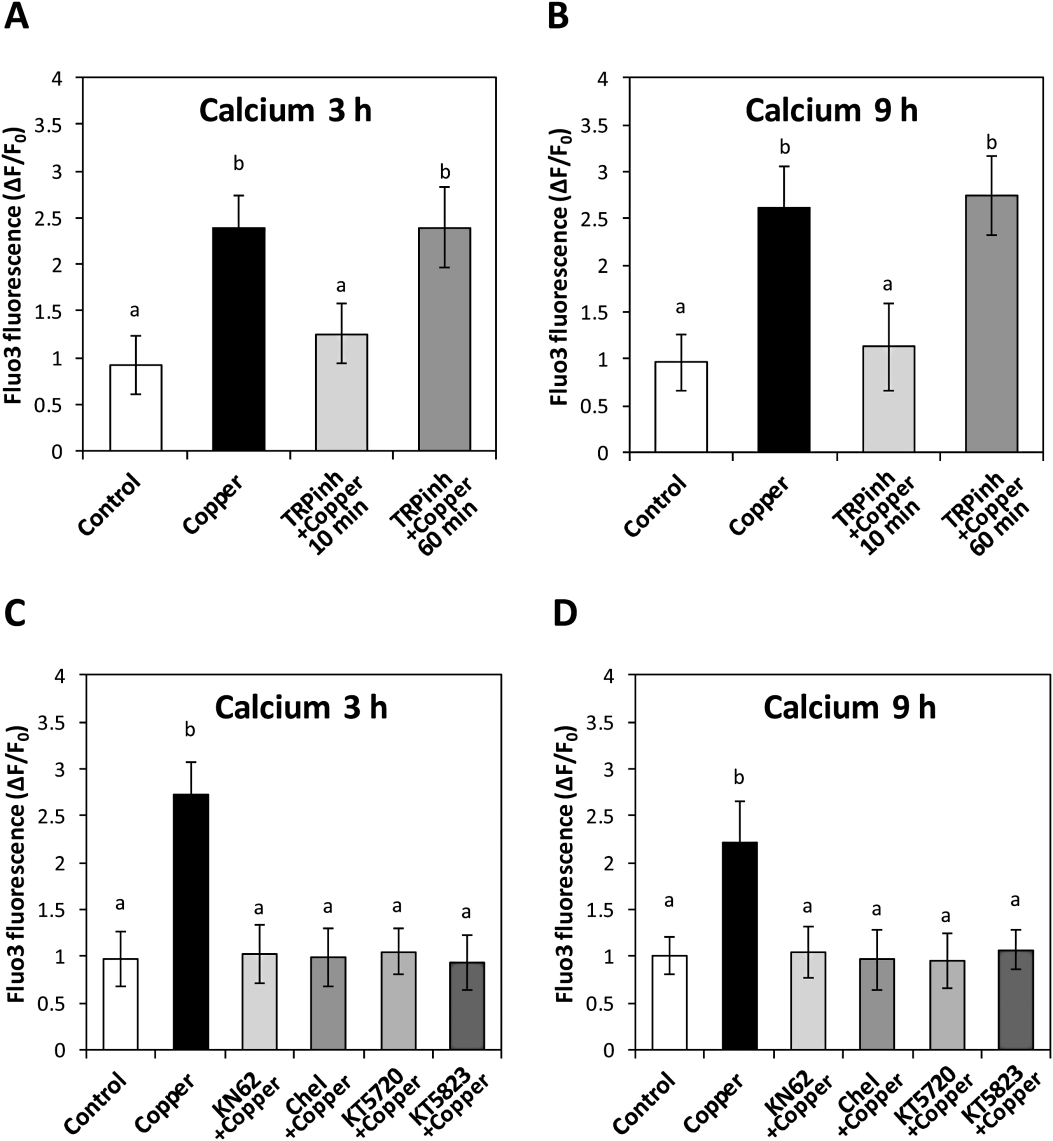
Relation of TRP channels activation with VDCC activation and protein kinases involved in this event. To address the role of TRPs in intracellular calcium in at 3 h (A) and 9 h (B) of copper exposure, treatments were: no copper addition (control); with 2.5 μM copper (copper); and with copper and 100 nM of TRP inhibitors (TRP inh; HC030031, ML204, M8B, capsazepin), added at 10 and 60 min of experiments. To study the role of protein kinases, the level of intracellular calcium in the alga under copper excess at 3 h (C) and 9 h (D) was assessed with the treatments: no copper addition (control); with 2.5 μM copper (copper); with copper and 100 nM KT5823; with copper and 100 nM chelerythrine (Chel); with copper and 100 nM KT5720; and with copper and 100 nM KN62. The level of intracellular calcium is expressed as the difference among initial and final fluorescence intensity of Fluo 3 normalized to initial intensity of Fluo3. Bars represent mean values of three independent experiments ± SD.

To observe whether copper-induced activation of TRPs participate in the activation of VDCCs, the alga was incubated with a mixture of TRP inhibitors containing 100 nM of inhibitors of TRPA1, C4, M8, V1; calcium increases known to occur at 3 and 9 h were analyzed. The mixture of TRP antagonists completely inhibited the increase in intracellular calcium at 3 and 9 h when added at 10 min of copper exposure (Figs. 4A and 4B). In contrast, TRP antagonists did not inhibit calcium increases recorded at 3 and 9 h when added at 10 min of copper exposure (Figs. 4A and 4B). In order to detect whether VDCC activation requires protein kinases activation, the alga was incubated under copper excess and inhibitors of CaMK, PKC, PKA and PKG; known occurring increases in intracellular calcium at 3 and 9 h of copper exposure were observed. Inhibitors of CaMK, PKC, PKA and PKG completely inhibited intracellular calcium increases at 3 and 9 h (Figs. 4C and 4D).

### Copper-induced activation of TRPs and VDCCs are involve in expression of proteins with roles in metal chelation

**Figure 5:**
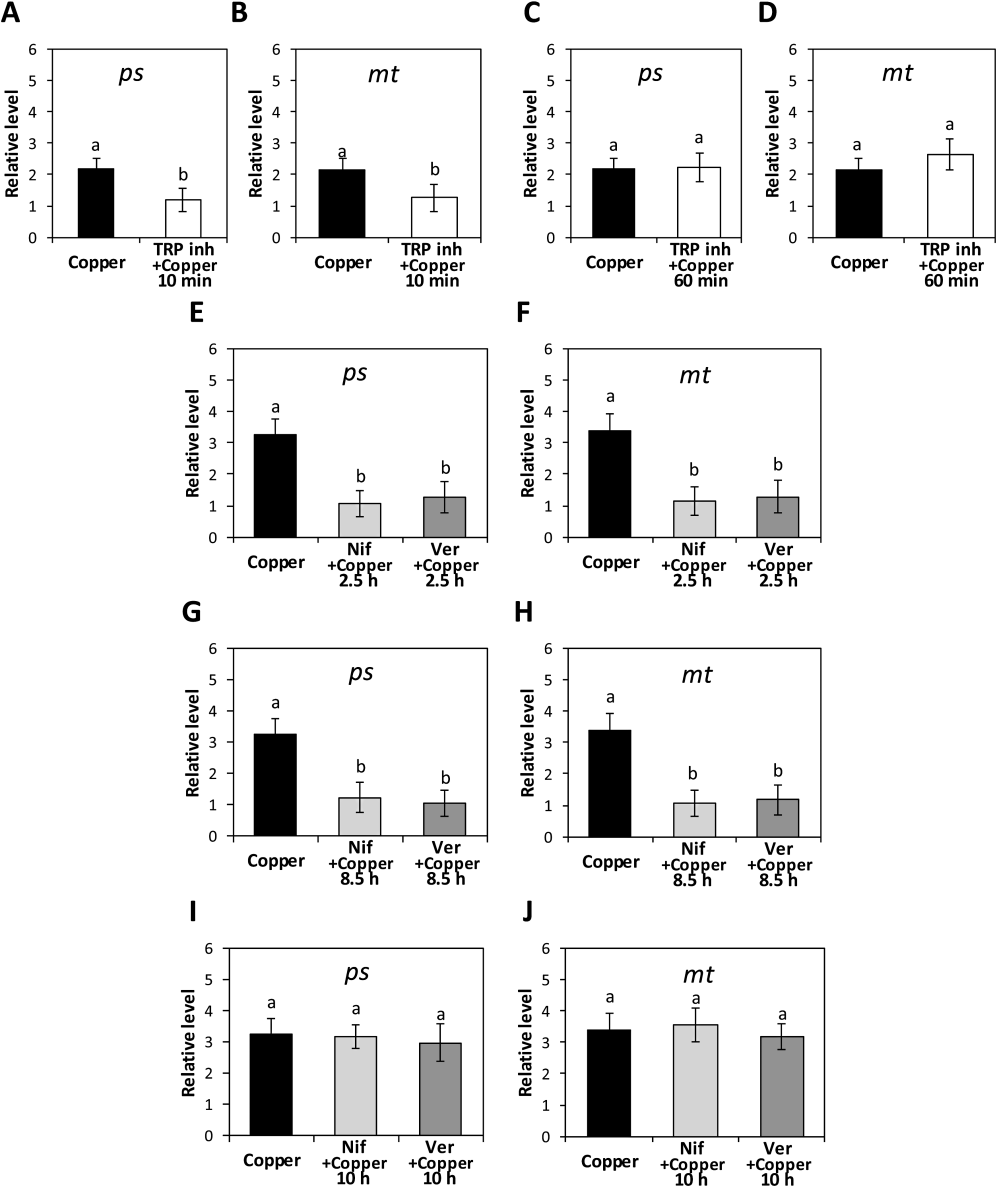
Transcriptional level of the heavy metal tolerance genes phytochelatin synthase (*ps*) and metallothionein (*mt*) in response to TRPs and VDCCs activation. Level of transcripts *ps* encoding phytochelatin synthase (A, C) and *mt* enconding metallothionein (B, D) in *E. siliculosus* cultivated with 2.5 μM copper and with 100 nM of a mixture of TRP inhibitors (TRP inh; HC030031, ML204, M8B, capsazepin), added at 10 min (A, B) and 60 min (C, D) of copper exposure. Levels of transcripts *ps* (E, G, I) and *mt* (F, H, J) in the alga cultivated with 250 nM nifedipine (Nif) and 250 nM verapamil (Ver), added at 2.5 h (E, F), 8.5 h (G, H) and 10 h (I, J) of copper exposure. The relative level of transcripts is expressed as 2^−ΔΔCT^. Bars represent the mean value of three independent experiments ± SD.

To address whether the increase in *ps* and *mt* transcript levels involve the activation of TRPs, the alga was incubated with a mixture of TRP inhibitors containing 100 nM of the inhibitors TRPA1, C4, M8, V1. The level of transcript of *ps* and *mt* were determined at 12 h of copper exposure. Transcript levels *ps* and *mt* decreased in response to TRP inhibitors added at 10 min of copper exposure (Figs. 5A and 5B), but not when these were incorporated at 60 min (Figs. 5C and 5D). To analyze the involvement of the activation of VDCCs on the increase in transcript levels, the alga was incubated with 250 nM of the VDCC inhibitors nifedipine and verapamil; these were added 10 min before the first VDCC activation (2.5 h), and 10 min before and after the second VDCC activation (8.5 and 10 h, respectively). Then, the level of transcripts was determined at 12 h of copper exposure. Transcript levels of *ps* and *mt* decreased in response to VDCC antagonists when these were added before the first VDCC activation (Figs. 5E and 5F), and before the second VDCC activation (Figs. 5G and 5H); the latter was not observed when the antagonists were added after the second VDCC activation (Figs. 5I and 5J).

## Discussion

### Copper-induced activation of VDCCs leads to extracellular calcium entry and intracellular calcium release from the ER

In this work, we showed that copper excess induced VDCCs activation at 3 and 9 h of exposure in *E. siliculosus*, allowing extracellular calcium entry and intracellular calcium release from the ER; these via activation of cADPR-, NAADP- and IP_3_-dependent channels. Thus, a calcium-induced calcium release (CICR) mechanism involving VDCC activation is operating in *E. siliculosus* in response to copper stress. A similar mechanism has been observed in the green macroalga *U. compressa,* which allows entry of extracellular calcium through VDCCs and the release of calcium from the ER at 1, 2 and 12 h of copper exposure; this process involves activation of cADR-, IP_3_- and NAADP-dependent calcium channels (González et al., 2012a, 2012b). In this regard, it is important to mention that cADPR-, IP_3_- and NAADP-dependent calcium channels were initially identified in the ER of animal cells; for instance, in skeletal, cardiac muscle, neurons and immune system cells (Lee, 1997; Laver, 2007). Different investigations also indicate that these channels may be also present in the ER of plants (Biswas et al., 1995; Muir & Sanders, 1996, 1997; Navazio et al., 2001). Considering that green and red algae are closely related organisms, and with terrestrial plants, but distant from brown algae (Cock et al., 2010), records on the existence of a CICR response in green algae, plants and animals, and now also in brown algae, demonstrates the universality of this mechanism to respond to environmental stimuli in eukaryotes.

### Copper-induced intracellular calcium increases activate CaMs, CBLs and CDPKs, leading to an increase in gene expression related to metal chelation

Here, we demonstrated that copper-induced activation of TRPs and VDCCs leads to increases in intracellular calcium transduced via CaMs, CBLs and CDPKs, triggering the increase in transcripts encoding proteins related to metal ions chelation; in this case, PCs and MTs. In this context, it is important to mention that key decoders of intracellular calcium increases are the calcium binding proteins CaMs, CBLs and CDPKs (Kim et al., 2009; Valmonte et al., 2014; Mao et al., 2016; Edel et al., 2017). CaMs, CBLs and CDPKs normally contain four calcium binding motifs having helix-loop-helix structure, designated EF-hands, which directly bind calcium. The binding of calcium induces a conformational change activating CaMs and CBLs that, in turn, bind to other effector proteins such as CaM-dependent kinases (CaMK) or CBL-interacting kinases (CIPKs); in contrast, calcium directly activates kinase activity in CDPKs (Kim et al., 2009; Valmonte et al., 2014; Edel et al., 2017). In plants, it has been observed that these protein kinases trigger transcription factors that activate or repress gene expression in order to tolerate abiotic and biotic stresses (Mao et al., 2016). Then, calcium increases observed in copper-stressed *E. siliculosus* are likely to be due to activation of TRP and VDCC channels; the latter since it was detected that TRPs allowed intracellular calcium increases at 13, 29, 39 and 51 min, whereas VDCC induced calcium increases at 3 and 9 h of copper exposure. Thus, calcium signature induced by copper in *E. siliculosus* is due to, at least in part, the activation of TRPs and VDCCs, which also lead to intracellular calcium increases. These may have differential intensity and temporality, and distinctly activate CaMs, CBLs and/or CDPKs that, in turn, potentially trigger gene expression of proteins involved in tolerance response.

### Copper-induced activation of TRPs and VDCCs are inter-connected events mediated by the activation of protein kinases, which also lead to activation of gene expression

Our results indicate that activation of VDCC require previous induction of TRPs and the activation of PKA, PKC, PKG and CaMK. In this sense, for instance, it has been determined that human vascular smooth muscle cells activate TRPC6 and TRPM4 under mechanic pressure, and that TRPC3 and TRPC6 are induced by diacylglycerol (Brayden et al., 2008). Moreover, it has been observed that TRPs activation can trigger VDCC of L-type, allowing calcium entry and calcium release from ER (Brayden et al., 2008). Thus, it is not surprising that a similar interdependent TRPs-VDCCs activation mediating a CICR response may occur in brown macroalgae, as recorded in *E. siliculosus* in this study. In this regard, TRPs-VDCCs interdependent activation has been recorded in the green macroalga *U. compressa* (Gómez et al., 2016). However, TRPs subunit composition and temporality of TRPs-VDCCs activation response to copper excess differ between *U. compressa* and *E. siliculosus*. In this investigation, we demonstrated that *E. siliculosus* under copper excess triggers VDCCs at 3 and 9 h of exposure. In contrast, VDCC in *U. compressa* are activated at 2, 3 and 12 h of copper exposure (González et al., 2010b, 2012a). Furthermore, in *E. siliculosus*, a TRPM8/V1 is activated at 13 min, a TRPV1 at 29 min, a TRPA1/V1 at 39 min, and a TRA1/C4 at 51 min of copper exposure (González et al., 2018), whereas in *U. compressa*, a TRPC5 is activated at 4 min, a TRPA1 at 8 min and a TRPV1 at 12 min (Gómez et al., 2015). Thus, brown macroalgae display a delayed activation of TRPs, but earlier induction of VDCC in response to copper stress, compared with green macroalgae. In addition, TRP subunit composition is more complex in *E. siliculosus* than in *U. compressa*. Moreover, protein kinase activation is required to trigger VDCC in *E. siliculosus,* involving the induction of CaMK, PKC, PKA and PKG. In this context, the activation of CaMK and PKC is probably due to calcium entry through TRPs, whereas PKA and PKG may be activated by other stimuli leading to the syntheses of cAMP and cGMP. Finally, the increase in gene expression requires the activation of calcium signaling proteins (see above). Since calcium is entering through TRPs and VDCCs, the information suggests that inhibition of TRP and VDCC may avoid the upregulation of proteins involved in metal chelation in *E. siliculosus* under copper excess.

## Conclusion

**Figure 6:**
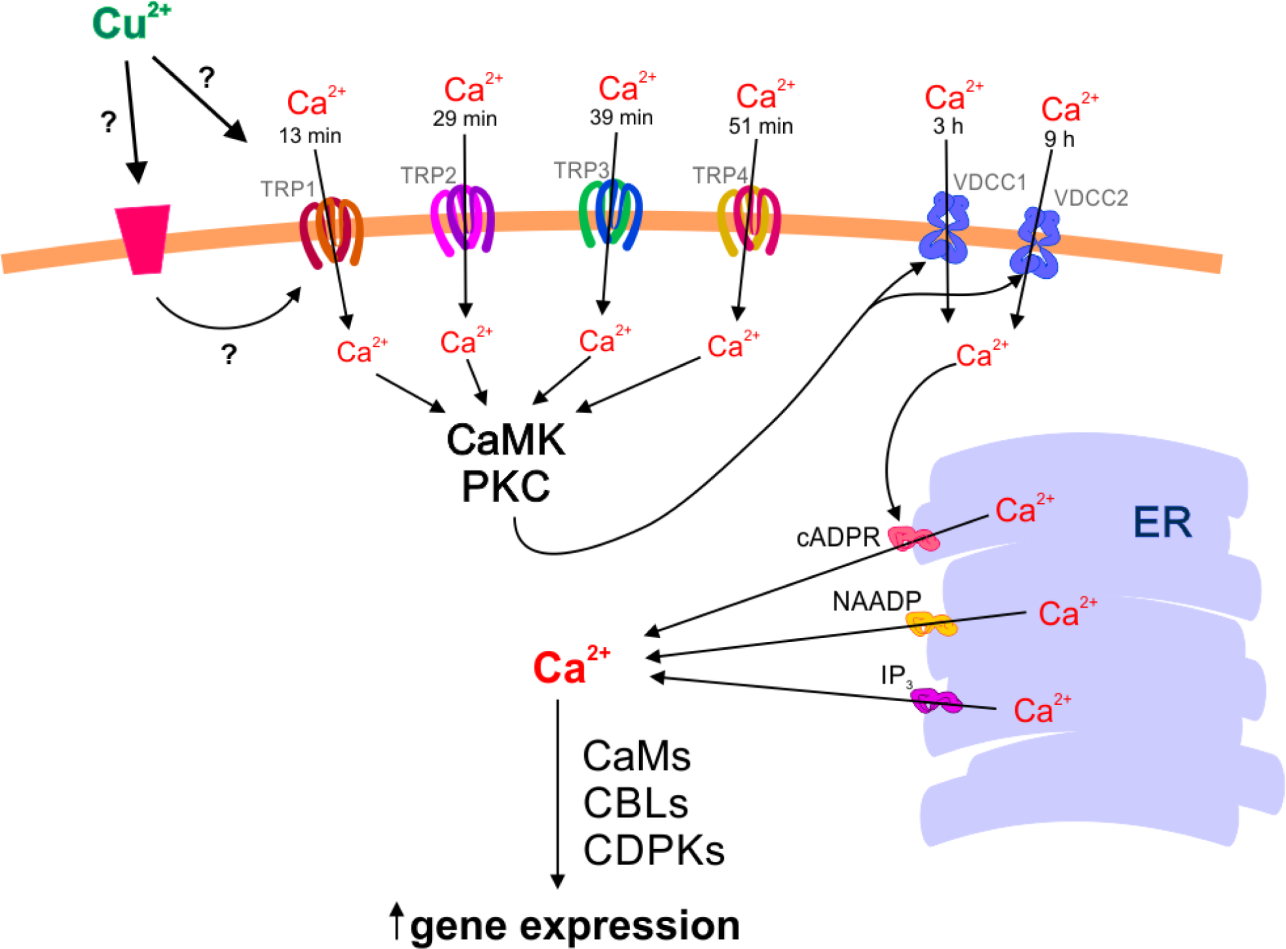
Model of calcium signaling activated by copper excess involving TRPs, VDCCs, intracellular calcium channels and calcium-dependent signaling proteins for the activation of tolerance genes. Copper ions (Cu^2+^) induced the activation of TRP channels at 13, 29, 39 and 51 min of copper exposure allowing extracellular calcium entry. This process activates CaMK and PKC leading to the induction of VDCC at 3 and 9 h of copper exposure, allowing extracellular calcium entry and intracellular calcium release from the endoplasmic reticulum (ER); the latter through cADPR-, NAADP- and IP_3_-dependent calcium channels. The increase in intracellular calcium activates calmodulins (CaMs), calcineurin B-like proteins (CBLs) and calcium-dependent protein kinases (CDPKs) that, in turn, lead to upregulation of proteins associated with metal chelation in *E. siliculosus*.

In this work, we demonstrated that copper excess induced the activation of TRP channels leading to extracellular calcium entry at 13, 29, 39 and 51 min, activating protein kinases that, in turn, trigger VDCCs at 3 and 9 h in *E. siliculosus*. This process allows extracellular calcium entry and intracellular calcium release via cAPR-, NAADP- and IP_3_-calcium channels located in the ER. Subsequently, the increase in intracellular calcium activates CaMs, CBLs and CDPKs mediating the increase of gene expression (see model in Fig. 6), in particular of proteins involved in the syntheses of the metal chelators PCs and MTs.

## Supplemental Information

10.7717/peerj.4556/supp-1 Supplemental Information 1 Images of *E. siliculosus* under copper and inhibitors treatments and verification of cell integrity. Confocal images of *E. siliculosus* loaded with Fluo 3 in control conditions without copper (A), cultivated with 2.5 μM copper for 9 h (B), cultivated with 2.5 μM copper for 12 h (C), preincubated with 250 nM verapamyl and cultivated with 2.5 μM copper for 9 h (D), preincubated with 250 nM nifedipine and cultivated with 2.5 μM copper for 9 h (E), and preincubated with 100 nM xestospongin C and cultivated with 2.5 μM copper for 9 h. Fluo 3 fluorescence is shown in green, chlorophylls autofluorescence in chloroplasts is shown in red. Scale bars are located at the bottom of each image.

10.7717/peerj.4556/supp-2 Supplemental Information 2 Statistical analysis for ps transcript levels at 6 h of copper exposure (Figs. 5A, 5C).

10.7717/peerj.4556/supp-3 Supplemental Information 3 Statistical analysis for ps transcript levels at 6 h of copper exposure (Figs. 5E, 5G, 5I).

10.7717/peerj.4556/supp-4 Supplemental Information 4 Statistical analysis for mt transcript levels at 6 h of copper exposure (Figs. 5B, 5D).

10.7717/peerj.4556/supp-5 Supplemental Information 5 Statistical analysis for mt transcript levels at 6 h of copper exposure (Figs. 5F, 5H, 5J).

## Additional Information and Declarations

### Data Availability

The following information was supplied regarding data availability: Figshare, Ectocarpus Es540 raw data: https://figshare.com/articles/Ectocarpus_Es540_Raw_data/5809797.
