# Supplementary figures and images for: Copper-induced activation of TRPs and VDCCs triggers a calcium signature response regulating gene expression in Ectocarpus siliculosus
Source: PeerJ. 2018 Apr 16;6:e4556. doi: 10.7717/peerj.4556 (PMC5907779; doi:10.7717/peerj.4556)

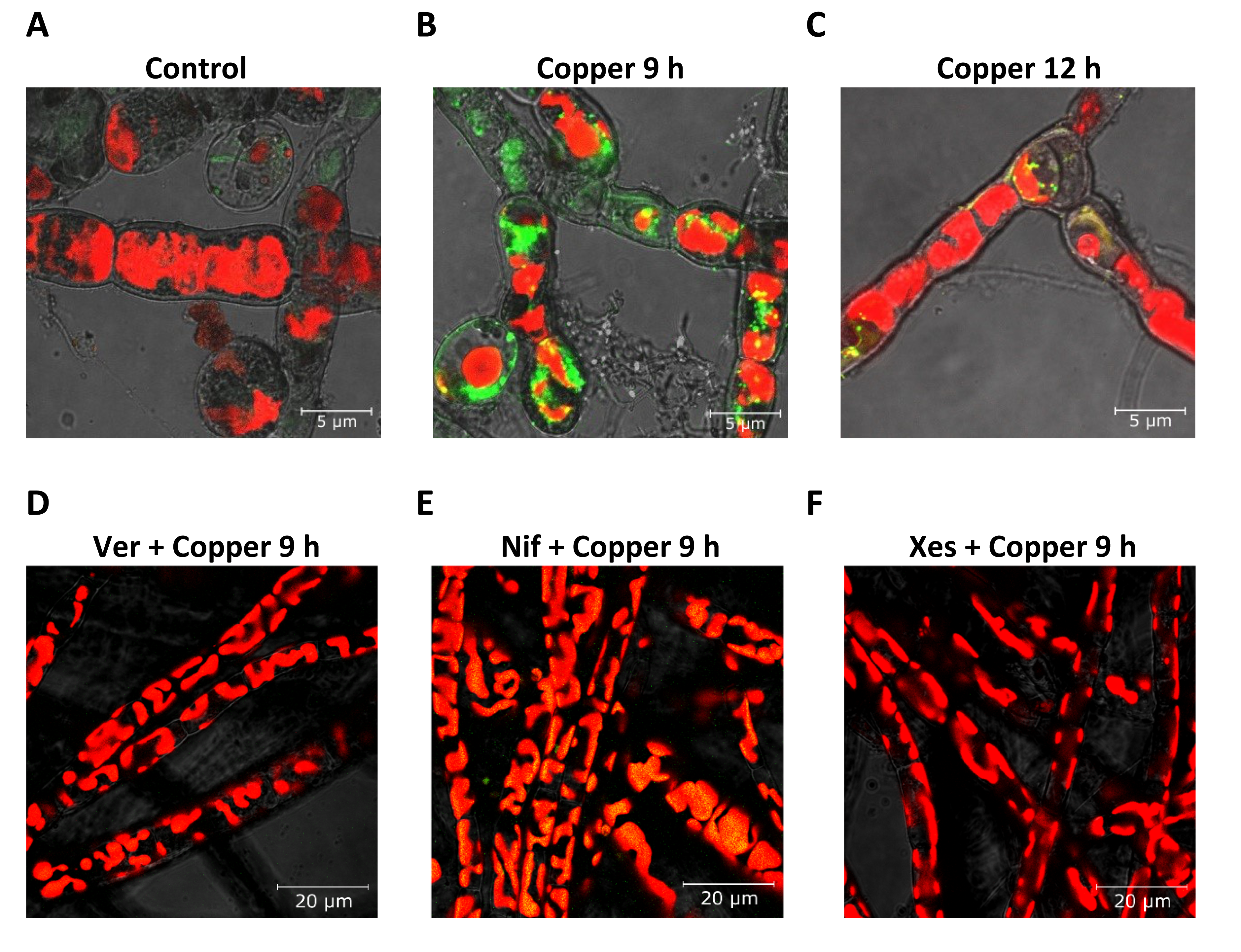

Supplement: Supplemental Information 1 — Confocal images of E. siliculosus loaded with Fluo 3 in control conditions without copper (A), cultivated with 2.5 μM copper for 9 h (B), cultivated with 2.5 μM copper for 12 h (C), preincubated with 250 nM verapamyl and cultivated with 2.5 μM copper for 9 h (D), preincubated with 250 nM nifedipine and cultivated with 2.5 μM copper for 9 h (E), and preincubated with 100 nM xestospongin C and cultivated with 2.5 μM copper for 9 h. Fluo 3 fluorescence is shown in green, chlorophylls autofluorescence in chloroplasts is shown in red. Scale bars are located at the bottom of each image. [file peerj-06-4556-s001.png]
